# Development and implementation of an integrated chronic disease model in South Africa: lessons in the management of change through improving the quality of clinical practice

**DOI:** 10.5334/ijic.1454

**Published:** 2015-10-12

**Authors:** Ozayr Haroon Mahomed, Shaidah Asmall

**Affiliations:** The Discipline of Public Health Medicine, University of KwaZulu Natal, Durban, South Africa; National Department of Health, Pretoria, South Africa

**Keywords:** integrated chronic disease management, continuous quality improvement, primary health care, HIV/AIDS, non-communicable diseases, case management

## Abstract

**Background:**

South Africa is facing a complex burden of disease arising from a combination of chronic infectious illness and non-communicable diseases. As the burden of chronic diseases (communicable and non-communicable) increases, providing affordable and effective care to the increasing numbers of chronic patients will be an immense challenge.

**Methods:**

The framework recommended by the Medical Research Council of the United Kingdom for the development and evaluation of complex health interventions was used to conceptualise the intervention. The breakthrough series was utilised for the implementation process. These two frameworks were embedded within the clinical practice improvement model that served as the overarching framework for the development and implementation of the model.

**Results:**

The Chronic Care Model was ideally suited to improve the facility component and patient experience; however, the deficiencies in other aspects of the health system building blocks necessitated a hybrid model. An integrated chronic disease management model using a health systems approach was initiated across 42 primary health care facilities. The interventions were implemented in a phased approach using learning sessions and action periods to introduce the planned and targeted changes.

**Conclusion:**

The implementation of the integrated chronic disease management model is feasible at primary care in South Africa provided that systemic challenges and change management are addressed during the implementation process.

## Introduction

South Africa has a two-tiered health care system [1], consisting of a private sector and a state-funded public sector that operate in parallel. The private health sector provides coverage to 17% of the population [1], despite spending an estimated 48% ($13 billion) of the national health expenditure [2]. The public health sector provides health services to approximately 83% (41 million) of the population [1] and receives its funding from the National Treasury. Funding for the public health care system has increased approximately 25% from $11 billion in 2010/2011 to $14.5 billion in 2013/2014 [3].

In 1994, with the dawn of democracy, a number of pro-equity reforms such as free maternal and child health care and free primary health care for all using the public health sector were introduced. South Africa adopted the district health system with primary health care as the vehicle for health service delivery [4]. To promote decentralisation and to build the district-based system, transformation focused on the organisational structure, authority and organisation of local services at a management level [5].

These structural changes were supported by a range of reconstruction and development initiatives such as infrastructural development targeting increased access to water and electrification; the comprehensive extension of social welfare grants to previously disadvantaged populations; and a national school nutrition programme [5].

However, despite the implementation of various initiatives aimed at strengthening primary health care, the impact at the population level has been limited.

These less than favourable outcomes are due to multiple factors that can be grouped under two broad categories namely; health system deficiencies and the protracted and complicated disease burden. The health system deficiencies include amongst others; inadequate and inappropriately trained health care personnel, inequitable distribution of health care personnel, inefficient and inequitable resource allocation (financial and equipment), multiple legacy health information systems that do not provide timeous information, a curative-oriented health service; and deficiencies in managerial capacity and health system leadership at all levels that have served to further perpetuate the complicated burden of disease [6].

The decline in life expectancy over the last decade is due to the emergence of infectious and non-communicable diseases, occurring alongside the high incidence of childhood diarrhoea and malnutrition as well as high levels of violence and accidents [7].

The under-5 mortality increased from 38 to 67 deaths per 1000 live births between 1998 and 2006. Between 2007 and 2010, the under-5 mortality declined to a level of 53 deaths per 1000 live births [8]. Infant mortality rate, increased from 26 to 48 infant deaths per 1000 live births between the period 1998 to 2007. By 2010, the infant mortality rate was approximately 38 deaths per 1000 live births [8]. Due to the increased mortality in early life, the life expectancy declined to 51.6 years for both sexes in 2005, but has increased to 58.7 years in 2012 [8].

According to the revised mortality and population estimates for South Africa (2000), HIV & AIDS accounted for almost 25.5% of deaths, chronic diseases of lifestyle were responsible for almost 40.8% of all deaths, other infectious diseases accounted for 22.2% of all deaths and injury-related causes accounted for 11.5% of all deaths [9].

Non-communicable diseases were the highest contributor to the mortality (40.8%) and to the disability-adjusted life years (33%) and the third most significant contributor to year’s life lost (22.8%) [9] in 2000. The World Health Organisation estimated that non-communicable diseases caused 28% of the total burden of disease measured by disability-adjusted life years in South Africa in 2004. Cardiovascular diseases (5.8%), diabetes mellitus (1.3%), respiratory diseases (2.6%) and cancers (2.5%) together contributed to 12% of the overall disease burden [10].

Despite the initial tardiness in implementing antiretroviral treatment for patients with HIV & AIDS, today South Africa has the largest antiretroviral therapy programme in the world with approximately 2.7 million patients receiving antiretroviral therapy in 2014 [11]. Of the patients receiving antiretroviral therapy, 85% were receiving antiretroviral therapy through the public health sector, 11% were receiving antiretroviral therapy through disease management programmes in the private sector and the remaining 4% were receiving antiretroviral therapy through community treatment programmes run by non-governmental organisation.

The unprecedented roll out of antiretroviral treatment has transformed HIV & AIDS into a chronic disease, as people with HIV are living longer and ageing, and are developing non-HIV-related chronic conditions similar to the rest of the population. Some non-communicable diseases are related to HIV infection itself and to the side effects of some of the medicines used to treat HIV infection [12].

As the burden of chronic diseases (both communicable and non-communicable diseases) increases providing affordable and effective care to the often large and increasing numbers of people will be an immense challenge. The current evidence suggests that South Africa’s response to non-communicable diseases has had little effect due to the rising incidence of deaths from non-communicable diseases in rural areas and the increasing pressure on health care services from acute and chronic diseases [13].

In addition, the poor and vulnerable population are exposed to an increased burden of risk factors associated with chronic communicable diseases such as HIV and tuberculosis as well as non-communicable diseases. This increased exposure to risk factors coupled with the failure of the health system to respond adequately through appropriate prevention, education and management of chronic diseases compound the consequences and costs associated with chronic diseases especially on the poor and vulnerable.

Providing affordable and effective care to the often large and increasing numbers of people is already an immense challenge but as the burden of chronic diseases both (communicable and non-communicable diseases) increases, this will become an even bigger problem requiring a different and innovative approach. The changing disease patterns requires a reorientation of the health system that will provide a comprehensive, effective and appropriate service for chronic, long-term care, while maintaining and improving the capacity of acute care services.

### Aim

**Figure 1.**
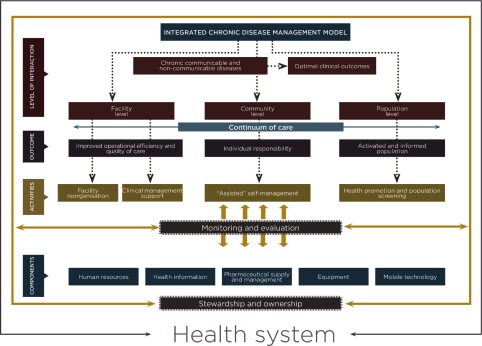
Integrated chronic disease management model for South Africa (Source: National Department of Health).

The aim of this paper is to describe the development and implementation of the integrated chronic disease management model for South Africa (Figure 1) [14]. We first developed the model and then implemented it, whilst simultaneously assessing its impact on operational efficiency, quality of care and sustainability using implementation research. The results of the implementation research are reported elsewhere.

## Methods

### Setting and structure

The South African public health sector follows a hierarchical system. Primary health care clinics are the first port-of-call for patients, and are meant to be preventative and patient-empowering focusing on maintaining a productive and healthy individual within the community, whilst simultaneously diagnosing and treating minor ailments. Services offered at the primary health care clinics are free of charge. Professional nurses who have received an additional primary health care training usually render the services. Medical practitioners visit the facilities on a rotational basis.

There are approximately 3100 primary health care clinics across the 11 provinces in South Africa. The population served by each of the clinics vary from 2500 to approximately 20,000, with an average of 12,000 people served per clinic. District hospitals play a central role between the primary health care clinics, community health centres, regional and tertiary hospitals.

The study was conducted across 37 primary health care clinics located in the three districts – 8 in Dr Kenneth Kaunda District, North West Province, 14 in West Rand Health District, Gauteng Province and 15 in Bushbuckridge sub-district within the Ehlanzeni District, Mpumalanga Province.

Dr Kenneth Kaunda District has a population of approximately 807,000 people. Health services are delivered by 1 regional; 3 district hospitals; 9 community health centres; 27 clinics; 6 satellite clinics and 2 mobile health service units.

The West Rand Health District has a population of approximately 900,000 people. The West Rand District Municipality has a total of 60 health facilities comprising 1 regional hospital, 2 district hospitals, 4 community health care centres and 39 primary health care clinics.

Bushbuckridge sub-district has an estimated population of 509,967. Three district hospitals, 2 community health care centres, 36 operational clinics and 5 mobile clinics form the platform to deliver health services.

The study was initiated in April 2010 with the implementation phase spanning the period between January 2011 and January 2013. The quasi-experimental study was conducted between December 2010 and October 2014.

### Approach and methods

Best practice is to develop interventions systematically, using the best available evidence and appropriate theory, then to test them using a carefully phased approach, and moving on to an exploratory and then a definitive evaluation [15]. Systematic improvement requires recognition of the systems and processes of the service being provided [16].

**Figure 2.**
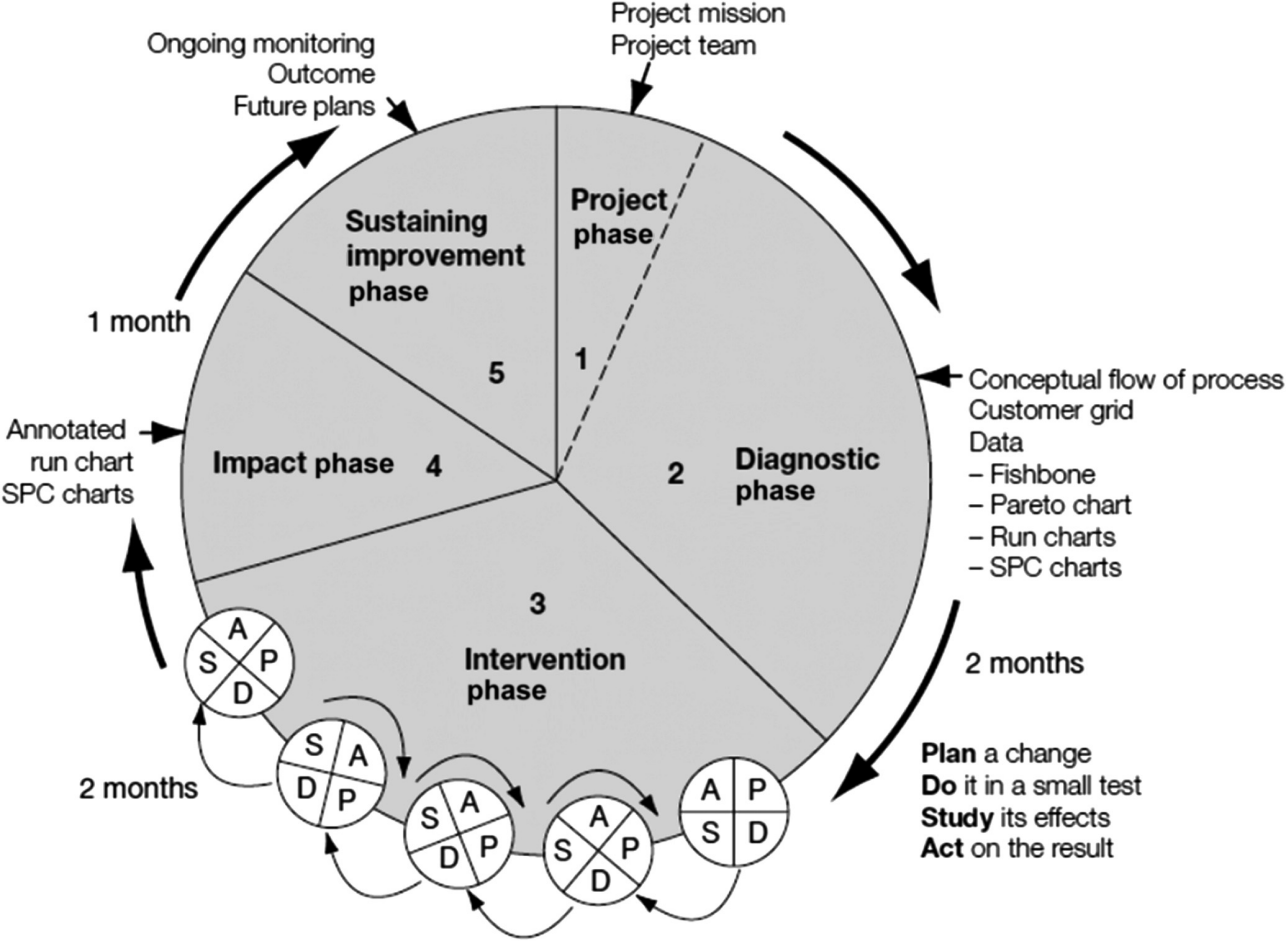
Clinical practice improvement model.

We utilised the clinical practice improvement model for continuous quality improvement [16] as the overarching framework for the development and implementation of the model (Figure 2). In developing the intervention, we utilised the framework proposed by the Medical Research Council of the United Kingdom in 2000 [17] and updated in 2008 [15] for the development and evaluation of complex health interventions. The breakthrough series as proposed for the Institute for Health improvement was used during the implementation process [18].

The five recognised steps in the clinical practice improvement process are (Figure 2):

1. Project phase: The project phase includes the Identification of the process/problem that requires improvement, forming teams, determining the aim of the intervention, deciding on the baseline data to be collected and the facilities to be enrolled.
2. Diagnostic Phase: the diagnostic phase involves collecting and analysing quantitative and qualitative data on the process being investigated to establish the causes of, and potential solutions to the problem.
3. Intervention phase (i) this phase speaks to Phase 1 of the Medical Research Council framework and includes identifying the evidence base and intervention components developing appropriate theory; modelling of outcome and process; identification of any incentives or barriers to intervention components [15].
  - Intervention phase (ii) – Implementation-The clinical practice improvement model is based on a ‘trial and learning’ approach to improvement-the Plan, Do Study Act cycle. This is consistent with Phase II of the MRC framework that speaks to: Feasibility and piloting of components, processes and procedures identified in Phase I; adaptation and modification to ensure optimal effect.
4. Impact phase involves the measuring and recording the effect of any changes.
5. Sustainability and improvement phase – Once improvements have been implemented, mechanisms need to be established to sustain the improvement. This may involve: standardisation of existing systems and processes for performing work activities; Documentation of associated policies, procedures, protocols and guidelines; Measurement and review to ensure that the change becomes part of the routine practice; Training and education of staff.

It is also important that continuous monitoring and the evaluation of long-term outcomes are built in to the system to allow for future improvements.

### Ethics

The study was approved by the National Department of Health. Ethics approval was obtained from the Bio-ethics and Research Committee of the University of KwaZulu Natal (BREC 423/13).

## Results

The present paper reports the application of the different phases of the clinical practice improvement model in developing and implementing the integrated chronic disease management model at primary care level. The result of the measurements conducted is reported elsewhere.

### Phase I – project phase

This phase provides the business case for change, faculty recruitment, enrolment of participating organisations and teams, determining the aim of the intervention, deciding on the baseline data to be collected and the facilities to be enrolled.

#### Case for change

In 2009, The Government of South Africa adopted an outcomes-based approach for its programme of action between 2010 and 2014. For the health sector, the priority is to improve the health status of the entire population [19]. The strategy of the National Department of Health to achieve the above outcome is encapsulated by the following four outputs of the National Service Delivery Agreement; increasing life expectancy; decreasing maternal and child mortality; combating HIV and AIDS and decreasing the burden of tuberculosis and strengthening health system effectiveness [20].

A renewed focus has been placed on strengthening the management of chronic conditions to address the output of increasing life expectancy. The proposed strategies to target chronic diseases includes the re-organising and improving the functioning of clinical services with the extension of care of all chronic diseases (both communicable and non-communicable) into communities through an integrated approach.

**Figure 3.**
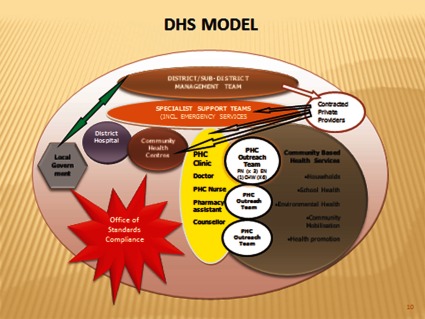
Primary health care re-engineering framework based on district health model.

The re-engineering of the health care system towards the primary health care approach has been adopted as a mechanism for improving the health systems effectiveness [21] and achieving universal coverage (Figure 3).

The primary health care re-engineering approach consists of three streams namely:

1. District-based clinical specialist teams (principal obstetrics and gynaecology, paediatrics, family physician, advanced midwife and a primary care-trained nurse) with an initial focus on improving maternal and child health.
2. A ward-based outreach team for each electoral ward; consisting of Professional Nurses; Enrolled Nurses and Community Health Workers in different wards across the country. The focus will be on health promotion, disease prevention and referral for curative care, to improve health outcomes.
3. An integrated school health team that will provide screening, education and preventive services at schools.

#### Faculty recruitment

A senior technical advisor was seconded to the National Department of Health to improve the quality of care and operational efficiency at primary care facilities. A public health medicine specialist (O.M.) was contracted to provide technical expertise for the refinement, implementation and monitoring of the outputs of such an implementation.

#### Enrolment of participating organisation

The non-communicable diseases cluster at the National Department of Health was the main sponsor of the project. Three districts located in three different provinces in South Africa that were to serve as the initiating sites were conveniently selected. These sites included rural and urban areas that provided care for mainly poor people. A consultative meeting was convened in each province with executive or senior management to identify the participating district that would serve as the initiating district. This was followed by an initiating meeting at each district with the district management team, to present the conceptual framework as well as request the district to select the initiating sites and then to convene a district meeting with all the selected sites.

At Bushbuckridge sub-district in Mpumalanga, 17 facilities were selected as initiation sites for the integrated chronic disease management from 5 local areas. Fourteen of the facilities can be classified as rural facilities, with access from the main arterial road to the facility being through a gravel/sand road.

Ten facilities were selected for the initiation of the integrated chronic disease management in Dr Kenneth Kaunda district. Three facilities are classified as: community health centres and seven facilities are primary health care clinic. Two of the facilities are urban facilities situated in a built-up environment whilst the other eight facilities are semi-urban situated in informal settlements or townships.

In West Rand Health district, 15 facilities were selected for the initiation of the integrated chronic disease management. Three facilities are classified as community health centres and 12 facilities are primary health care clinics. Thirteen facilities are semi-urban and situated in informal settlements or townships and two facilities are rural.

#### Establishment of teams

A district team was convened for each district. The district team consisted of the district manager or designate as the task team leader, district health services manager, district clinical manager, district pharmacist, district health information manager, district HIV/AIDS coordinator, district chronic and mental care coordinator, district tuberculosis manager, the local area/sub-district managers and the operational managers from each of the selected facilities as well as the Provincial HIV/AIDS, Tuberculosis, non-communicable diseases and mental health coordinators. The main role of the district task team was to champion the project; to interact with key officials in the service delivery chain to overcome systemic limitations, conduct the baseline assessments, work with the operational managers in developing quality improvement plans, provide monitoring and supportive supervision and provide report backs at the task team meetings.

### Phase II – diagnostic phase

The diagnostic phase involves collecting and analysing quantitative and qualitative data on the process being investigated to establish the causes of, and potential solutions to, the problem.

The district teams were capacitated on the methodology for conducting a baseline assessment of the facilities and were allocated to the facilities for oversight and conducting the baseline assessments. In addition to the task team members, the public health specialist and/or the senior technical advisor visited each of the facilities. The baseline assessment consisted of an interview with the operational manager to obtain contextual information of the services offered at the facility, a facility walk through to identify the patient process flow, development of a facility sketch map showing all service and administrative areas, review of the facility health information collected for the district health information system and the HIV/AIDS grant and a review of the number of health care workers and their skills. The baseline assessment was conducted between April 2011 and November 2011 across the 42 facilities.

The findings of the baseline assessment indicated the nature of the challenges were systemic and included the following amongst others:

- Inefficient processes of care characterised by vertical nature of services – Clinical services were delivered in designated consulting rooms for specific programmes.
- Services were not offered daily. There were specific days for consulting chronic non-communicable disease patients.
- Inefficient process flow at all facilities – All patients wait in one area for vital signs monitoring – resulting in bottlenecks and extending patients’ waiting times, No patient scheduling mechanism in place – Patients were given only return dates for follow-up – thus inappropriate staff allocation and no mechanism for tracking defaulters.
- Variation in the availability of clinical records for patients, with only 20 of the 40 facilities (48%) having facility-based patients’ records for acute and chronic patients. At facilities where there are no records for acute cases, patients are required to carry their own patient record books (exercise books).
- Poor quality of medical record keeping with inadequate recording of examination findings
- Specialised human resource nurses consulting patients for specific condition
- Data driven service, where there is emphasis on data collection but little attention to accuracy and completeness of the data. There is a bias towards collection of accurate data for the HIV programme through utilisation of specially designed data collection tools.
- Variation in supply and availability of medication and essential equipment.

The results of the baseline assessment were then presented to each district at a learning session. A quality improvement template was provided to each facility by the facilitators and the district task team and facility task teams were then asked to brainstorm the root causes of the deficiencies identified in the baseline assessment.

### Phase III – intervention phase

Phase 1 of the Medical Research Council of United Kingdom for the implementation of complex health interventions guided the development of the intervention. The main steps include identifying the evidence base and intervention components, developing appropriate theory; modelling of outcome and process; identification of any incentives or barriers to intervention components.

Having mapped the process and identified the nature of the problems associated with the treatment and management of chronic patients, the National, Provincial and district task team concurred with the Lancet series and noted that an overhaul in the approach to the management of chronic patients was required. This required a complex health intervention directed at each of the health system building blocks and along the continuum of care to improve the outcomes for patients with chronic diseases. This laid the foundation for the development and implementation of an integrated chronic disease management model.

A review of the evidence for the design and effectiveness of Chronic Care Models was conducted. A systematic review of published articles between January 2000 and March 2008 that evaluated Chronic Care Model-based intervention showed that the implementation of multiple elements were associated with better quality of care. A meta-analysis showed that four elements of the Chronic Care Model (Delivery System Design, Self-Management Support, Decision Support and Clinical Information Systems) were associated with better outcomes and processes [22]. The results of studies vary as to which of the individual Chronic Care Model elements are the most effective. However, delivery system redesign and support for self-care have been shown to be the most powerful interventions [23].

The most compelling evidence supporting the effectiveness of Chronic Care Model on improving patient outcomes have been demonstrated through a randomised controlled trial conducted across 311 Danish practices with 474 general practitioners (243 in intervention group and 231 in comparison group). The findings of this study showed that there was a reduction in fasting plasma glucose concentration, HbA1c, systolic blood pressure and cholesterol amongst type 2 diabetic patients to levels that have been shown to reduce diabetic complications [24].

In 2002, Médecins Sans Frontières and the Cambodian Ministry of Health established chronic disease clinics to integrate HIV/AIDS care with the management of diabetes and hypertension in two provincial capitals, Takeo and Siem Reap. The findings of this study showed positive outcomes on patients at 24 months of treatment. The median CD4 count of HIV patients on hghly active antiretroviral therapy rose from 53 cells/mm^3^ at baseline to 218 cells/mm^3^ at month 12 and 316-cells/ mm^3^ at month 24 of treatment. Just over half (57%) of the patients had an HbA1c below or equal to 9%. Of all hypertension patients who were on regular drug therapy for more than 6 months, 68% had reached blood pressures equal to or below the target of 160/90 mmHg [25].

We explored the literature for an appropriate model for implementing change at primary care clinics. The theoretical framework “How to Lead Improvement for PPRNet-TRIP” that was developed to implement change in primary care practice [26] was utilised as the model to initiate and implement the change required. This model consists of seven concepts (vision with clear goals; team involvement; enhance communication systems; develop staff knowledge; take small steps; assimilate electronic medical record into clinical practice to maximise clinical effectiveness; and feedback within a culture of improvement) and is driven by strong leadership.

We involved the district management team and the operational managers of each facility in each of the decisions. The results of the situation analysis were presented to a plenary session. A facilitative process was conducted to perform a root cause analysis of each of the deficiencies highlighted. The facilitators then grouped the main issues that were raised by the participants and classified them into the components of the health system building blocks. This was followed by a review session in which the various Chronic Care Models and their respective components were presented to the team. Although, the Chronic Care Model was ideally suited to improve the facility component and patient experience, the deficiencies in other aspects of the health system building blocks required attention to ensure the sustainability of the model and therefore a hybrid model was adopted by the district management teams. This model was then approved by the National Department of Health for initiation. The followings are the implementation steps:

#### Organisation of health care

Lean thinking principles were applied to reduce the wastage within the system. An appointment scheduling system that was patient centric was introduced to evenly distribute patient load. The administrative component of patients’ records retrieval and filing was re-organised to a single clinical record per patient that was stored according to a unique identifier, rather than disease condition. A combination of an enhanced filing system and appointment scheduling system facilitated the pre-retrieval of patient records.

#### Delivery systems re-design

Patients with chronic communicable diseases and non-communicable diseases were integrated into a single service. A designated vital sign station was introduced for chronic patients. Specific consulting rooms for chronic patients were designated based on the patients'load. Patient medication was pre-dispensed and available from the chronic consultation rooms. Stable patients were provided with two–three months of medication depending on availability. Patients that were stable and showed no signs of complications were down referred to the ward-based outreach team for self-support and management.

#### Clinical management support

Primary Care 101–a user-friendly algorithmic-based guide based on the latest South Africa public sector guidelines for primary care was made available for each consulting room at the facilities. An educational outreach programme based on the train the trainer’s model was implemented to capacitate professional nurses with appropriate skills to diagnose and manage patients in an integrated manner.

#### Clinical information system

Accurate record keeping is essential to promote continuity of care and better patient monitoring. A structured clinical record was designed and implemented to allow for accurate clinical record keeping and patient monitoring for a 12-month period.

#### Assisted self-management support

We intended on promoting patient empowerment and ownership for their own health by providing access to point of care testing of blood sugar and blood pressure at the patient residence through the services of the ward-based outreach team. In addition, patients were to be provided with health promotion guides and talks at their residence.

The sustainability of these facility-based interventions were dependent on an optimally function health system. We used this opportunity to strengthen the health system across various programmes (diagonal health system strengthening).

#### Human resources

We aimed for optimal utilisation of scare resources by providing an outreach-based training programme that equipped all medical practitioners and professional nurses on management of all common conditions consulted at primary care. This allowed for integration of health professionals to manage all conditions rather than specific programmes. Second, tasks that could be delegated to a lower level of health cadre were identified and the necessary training was provided to the lower level cadre.

#### Medicine supply and management

A concerted effort was made in reducing medication stock outs through improving stock management. Through accurate data collection, it was possible to calculate the number of clients with the specific disease conditions being consulted at the facility. This allowed for forecasting of medication needs. In addition, training on updating of stock cards was provided.

#### Equipment supply

The availability of good quality essential medical equipment that is well maintained is fundamental to the implementation of the integrated chronic disease management. Although, we could not provide this equipment, we conducted an international review and identified essential equipment required for each point of service. We conducted an audit to identify the requirements for each facility and provided this to the district.

#### Health information

Accurate data is required for planning. We introduced a data collection tool that combined the data being collected through various platforms. We produced a simple algorithm that allowed operational managers to utilise the information for planning of human resources and patient scheduling. In addition, the information generated allowed the operational managers to identify diseases that required better management from both a provider and patient’s perspective.

#### Leadership and advocacy

We used this as a platform to involve the community in taking responsibility for their own health. The clinic committees served as the primary role of communicating the changes within the facilities. In addition, age-specific support and adherence groups were launched within the facility and community.

### Phase IV a: implementation process

The interventions were implemented in a phased approach using learning sessions and action periods of the breakthrough series to introduce the planned and targeted changes. The district team and operational managers were capacitated on the various steps for the implementation of the integrated chronic disease management model. The initial step was the selection of a facility-based champion that will be responsible for the day-to-day implementation of the activities. The champions were then trained on each step that needed to be followed for implementation. During the learning sessions, the champions worked in groups and developed detailed quality improvement plans with time frames. Each facility was allowed the liberty to customise the interventions to suite their specific facility although the implementations of all the elements were mandatory. An initial meeting with all categories of staff was called at each facility to brief and engage with them regarding the process. An operational team with designated roles and responsibilities from each category of personnel was convened. A period of 6–8 weeks was allowed for informing the patients and engaging with the clinic committees, local political leaders, traditional leaders and private practitioners.

Prior to finalising the health service delivery re-design phase, the senior technical advisor and public health medicine specialist visited each facility to ensure the process flow within the facility was efficient to identify any potential challenges. To ensure continuous support for the process, the local area managers/district task team conducted monthly facility supportive visits.

The senior technical advisor, public health medicine specialist, provincial managers and district managers on a quarterly basis conducted facility supportive supervisory visits. The main aims of the supportive visits were to explore and/or address any challenges encountered and, second, to assist the facilities in the implementation of its individual goals.

### Phase IV b: impact assessment

Phase IV– Impact and implementation phase– The impact of the changes should be measured in order to be the intervention has resulted in an improvement, and to provide the evidence required to justify permanent implementation of these changes. This is linked with the measurement and evaluation phase of the breakthrough series collaborative.

The impact assessment phase represents the study phase of the cycle. At the outset, the main aim was to improve the quality of care and improve the clinical outcomes of chronic patients at primary clinic level.

- A process evaluation was conducted to assess the implementation of the various components of the intervention.
- A cross-sectional study was conducted to determine patient and staff experiences with the integrated care.
- A quasi-experimental study was conducted prior to the intervention, 6, 12 and 24 months to determine the improvement in operational efficiency.
- Lot of quality assurance sampling methodology was used to determine whether there was an improvement in the quality of clinical records.
- A sustainability assessment was conducted two years post initiation of the model.

The results of these studies are reported elsewhere.

### Phase V a: improvement phase

The quarterly facility supportive visits and the results generated from the waiting time surveys and process evaluation, allowed us to identify facility-specific challenges, deficiencies in the understanding of the process by the district and facility teams as well as certain deficiencies in the newly applied and implemented tools. The facility-specific challenges and challenges with the understanding of the process were addressed through a group interaction during the support visits and escalation of health system challenges to the district, provincial and national department for consideration. The tools that were developed for the work activities were reviewed and modified to standard operating procedures.

### Phase V b: sustainability phase

In order to promote the sustainability of the initiative and decrease the reliance on external facilitators, a step-by-step manual was developed that provided easy to use guidelines for implementation of all components of the model. The manual was piloted and developed in conjunction with the key implementers within the three districts. Capacity-building workshops were conducted in the three districts to train the relevant managers on implementing, monitoring and sustaining the integrated chronic disease model.

## Discussion

An integrated approach to the management of chronic diseases derived from the Wagner’s Chronic Care Model [27] and World Health Organisation improved care for chronic conditions framework [28] was developed and modified with the explicit aim of ensuring an integrated response of communicable and non-communicable chronic diseases. The implementation of the integrated chronic disease model was “real world” and not an experimental design. The aim was to achieve a model for implementation that would be replicable without additional resources and the use of external facilitators. We incorporated various processes for implementation from the clinical practice improvement model, the Medical Research Council framework and the breakthrough series for the development and implementation of the integrated chronic disease management.

The successful implementation of the HIV/AIDS programme at clinic level in South Africa demonstrated that with appropriate planning and the introduction of simplified tools, health professionals were able to implement new programmes successfully. However, the integrated chronic disease management model went further than this by producing comprehensive and sustainable organisational and community change through a radical shift in the approach to service delivery. Key requirements for implementing and sustaining change are change management; project planning and the use of quality improvement tools [29], and these together with health service ownership were the central themes that resonated throughout the implementation process.

At the outset, we involved the provincial, district, local area and facility managers in the process and provided them with the opportunity to lead the process at the local level. The information generated from the initial baseline assessment and the subsequent learning session in developing the quality improvement plan, motivated both the operational managers and local area managers in taking responsibility for the changes. These learning sessions facilitated the sharing of ideas and best practices and served to enhance the motivation of the early innovators.

Despite every effort to take cognisance of and address potential barriers, the implementation of the integrated chronic disease management was dependent on the leadership capabilities of the operational managers. Many of the operational managers were appointed to their positions based on seniority and without any management and leadership training. This negatively affected the translation of the required activities at facility and community levels, as this required a fundamental shift from their normal functioning. In order to overcome this stumbling block and shift responsibility away from an already overwhelmed operational manager, we created the role for an “integrated chronic disease management model champion” that was responsible for implementing the integrated chronic disease management model in consultation with the operational manager. However, in a few facilities this served to increase inter-personal conflict.

Cross messages from the various programme managers and the reluctance of the designated HIV/AIDS nurses that initiated and managed only HIV/AIDS patients created some organisational resistance. In order to overcome the challenges, we invited all the programme managers to our learning sessions and articulated the benefits of working in an integrated manner and the anticipated improvement in the quality of care to the patients.

During the facility supportive supervision visits, we actively engaged with the staff at the facility and included all cadres of staff from the general assistants to the medical officers as well as the health care workers that were appointed specifically for the HIV/AIDS programme. During these interactive sessions, we allowed the staff to verbalise their understanding of the integrated chronic disease management and their feeling towards it. Much of the initial resistance was the result of misunderstanding of the roles and responsibilities of each staff member and the anticipated increased workload. In addition, the professional nurses in charge of initiating and managing patients initiated on antiretroviral therapy, expressed concerns that all professional nurses were not trained on antiretroviral therapy initiation. The project team, together with the district task team and relevant programme managers addressed the concerns of the staff, even though some of the HIV/AIDS programme staff were still reluctant to integrate.

Systemic challenges inherent in the health system negatively impacted on the smooth implementation of the integrated chronic disease management model. For example, although the facilities were prepared and commenced with implementing various activities of the model, the lack of adequate essential equipment required at the facility posed a major challenge. This challenge was cascaded to the highest level by the project implementing team, but the lack of financial resources and the procurement of poor quality equipment without a maintenance plan in place hindered the process.

Another limiting factor is that the health services is still very focused at a curative health service level, with inadequate emphasis on primary prevention, health promotion and empowering the communities and patients to take responsibility for their individual health. A limitation of the model developed is that these aspects were not sufficiently included; however, the development of a compendium for health promotion is now being developed and will be added. The roles of the community health worker as couriers of medication is still in the process of being defined and will be incorporated in the implementation model in the near future.

Measurement, evaluation and feedback are important in ensuring continuous improvement. A quasi-experimental study design using before and after measures was included as part of the implementation plan. The 42 clinics that were part of the initiation should be seen as part of the process of the development of the model rather than as clinics implementing a final product. The focus of the evaluation was on operational efficiency and quality improvement and the process lessons that have been learned and described here have become integral to the model that will be rolled out. The model needs to be evaluated using a cluster randomised control trial utilising internationally validated tools such as Patients Assessing their Chronic Illness Care [30] and the Assessment of Chronic Illness Care tool [30].

## Conclusion

The breakthrough series and the continuous quality improvement model provided important structure for the implementation of the integrated chronic disease management model at clinic level in South Africa. The implementation of the integrated chronic disease management model is feasible at primary care in South Africa provided that systemic challenges and appropriate change management is not neglected. Now that the model has been developed and refined through direct implementation and learning derived from this experience it is important that a prospective study be conducted to determine the sustainability of the integrated chronic disease management model implementation and impacts on overall service quality, clinical outcomes and cost effectiveness.

## Authors’ contributions

O.H.M. implemented the study and drafted the manuscript. S.A. was instrumental in conceptualisation and participation in the implementation. All authors have read and approved the final manuscript.
